# Bilateral Endoscopic Intracerebral Hemorrhage Evacuations at Two Separate Time Points: A Case Report

**DOI:** 10.7759/cureus.20613

**Published:** 2021-12-22

**Authors:** Guilherme Barros, Dominic Nistal, Michael L Martini, Christopher P Kellner, Michael R Levitt

**Affiliations:** 1 Department of Neurological Surgery, University of Washington, Seattle, USA; 2 Department of Neurosurgery, Icahn School of Medicine at Mount Sinai, New York City, USA; 3 Department of Neurosurgery, Mount Sinai Health System, New York City, USA

**Keywords:** case report, endoscopic evacuation, intraparenchymal hematoma, hemorrhagic stroke, intracerebral hemorrhage

## Abstract

In this case report, we describe bilateral endoscopic intracerebral hemorrhage (ICH) evacuations in patients presenting on temporally distinct occasions with separate, contralateral lesions. Two patients presented with spontaneous right-sided ICH and underwent endoscopic evacuations. Both patients achieved some degree of functional improvement postoperatively. Each patient then experienced a second ICH in the left hemisphere months later, and again underwent endoscopic evacuation of the contralateral lesion. Postoperatively, both patients faced significantly longer hospitalizations and severe drops in functional independence compared to the first surgery. Functional outcomes after contralateral endoscopic ICH evacuation may vary significantly, and bilateral disease portends a worse prognosis.

## Introduction

Intracerebral hemorrhage (ICH) is the most devastating form of stroke, associated with a one-year mortality rate of over 50% [1]. While surgical intervention for ICH has been trialed over the years without consistent success [2-5], recent technological developments have enabled the development of minimally invasive ICH evacuations techniques [6-10], which have shown beneficial long-term outcomes in certain populations after surgical intervention for ICH [8,11-13]. However, patients rarely present with two separate hemorrhagic events in both time and cortical locations. There is no currently published data investigating bilateral ICH evacuation in two distinct time points in patients. We describe two unique patients with initial right-sided ICHs, who then presented over a year later with subsequent, left-sided ICHs, both treated via endoscopic evacuation.

## Case presentation

The retrospective review was performed to identify patients who experienced two or more ICHs in different cerebral hemispheres treated with endoscopic evacuations at our institutions. Evacuations were performed according to previously described methods [12]. Informed consent was not requested or required by our Institutional Review Boards. This report is compliant with both institutions’ research requirements.

Case 1

**Figure 1:**
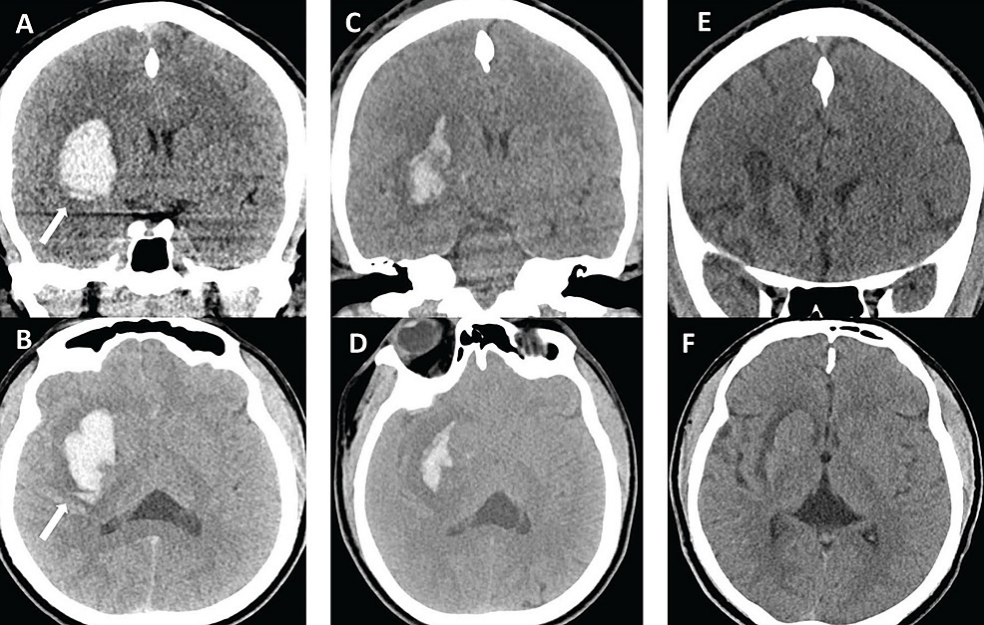
Case 1 - initial admission pre- and postprocedure and follow-up imaging. Preprocedure initial admission non-contrast coronal (A) and axial (B) head computed tomography (CT) demonstrates a 21.4 mL right basal ganglia hemorrhage (arrow). Postprocedure coronal (C) and axial (D) non-contrast CT demonstrated 44.4% evacuation with remaining hematoma measuring 11.9 mL. Coronal (E) and axial (F) follow-up imaging three months post hemorrhage shows resolution.

A 45-year-old male with hypertension presented with sudden onset left-sided weakness. On arrival, he was alert, oriented, and following commands with left hemiparesis and a National Institute of Health Stroke Scale (NIHSS) of 9. Non-contrast computed tomography (CT) head demonstrated a 21.4 mL (ABC/2 method [14-16]) right basal ganglia ICH causing 6mm midline shift and perilesional edema, without intraventricular hemorrhage (IVH) (Figures 1A, 1B). The neurologic exam remained stable, and CT angiogram (CTA) was negative for any abnormality. Due to the risk of progressive edema causing local mass effect and potential for worsening neurologic exam, surgical evacuation was offered. The patient underwent endoscopic evacuation on hospital day 2 [12]. Intraoperative CT demonstrated a 44.4% reduction of hematoma volume from 21.4 mL to 11.9 mL (Figures 1C, 1D). The patient was discharged home on postoperative day (POD) 5 with a modified Rankin Scale (mRS) of 2 (Figures 1E, 1F).

**Figure 2:**
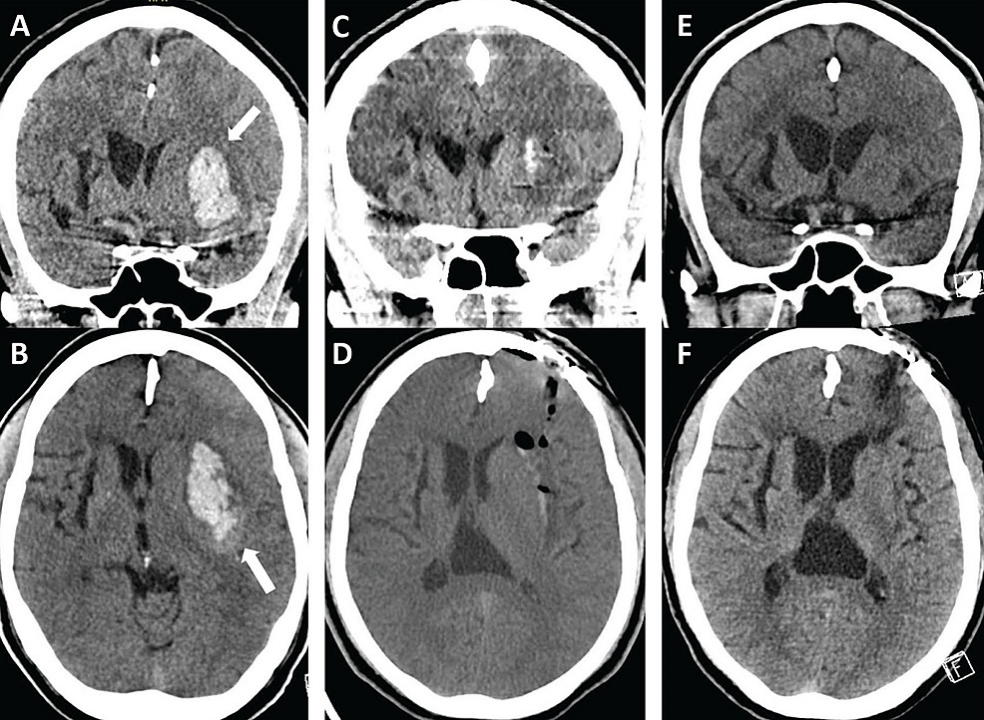
Case 1 - second admission pre- and postprocedure and follow-up imaging. Preprocedure second admission non-contrast coronal (A) and axial (B) head computed tomography (CT) demonstrate a 29.8 mL left basal ganglia hemorrhage (arrow).  Postprocedure coronal (C) and axial (D) non-contrast CT demonstrated near-complete evacuation, with a 95.0% evacuation rate and remaining hematoma measuring 1.5 mL. Coronal (E) and axial (F) follow-up imaging more than three months post hemorrhage show resolution and expected encephalomalacia.

The patient presented 38 months later with acute aphasia, right-sided weakness, and altered mental status. Neurological exam demonstrated somnolence with eyes opening only to noxious stimuli, localizing to painful stimuli with his left upper extremity, withdrawing his left lower extremity to painful stimuli, and right-sided hemiplegia for an NIHSS of 23. CT demonstrated a 29.8 mL left basal ganglia ICH without IVH, producing 7 mm of midline shift (Figures 2A, 2B). The patient’s exam remained stable, and CTA was negative for vascular abnormalities. The patient underwent another endoscopic evacuation on hospital day 1. Postoperative hematoma volume was reduced 95%, from 29.8 mL to 1.5 mL (Figures 2C, 2D). Postoperatively, the patient’s hospital course was complicated by acute respiratory failure with prolonged intubation and percutaneous endoscopic gastrostomy placement, with eventual admission to inpatient rehabilitation. His six-month mRS was 4 (Figures 2E, 2F).

Case 2

**Figure 3:**
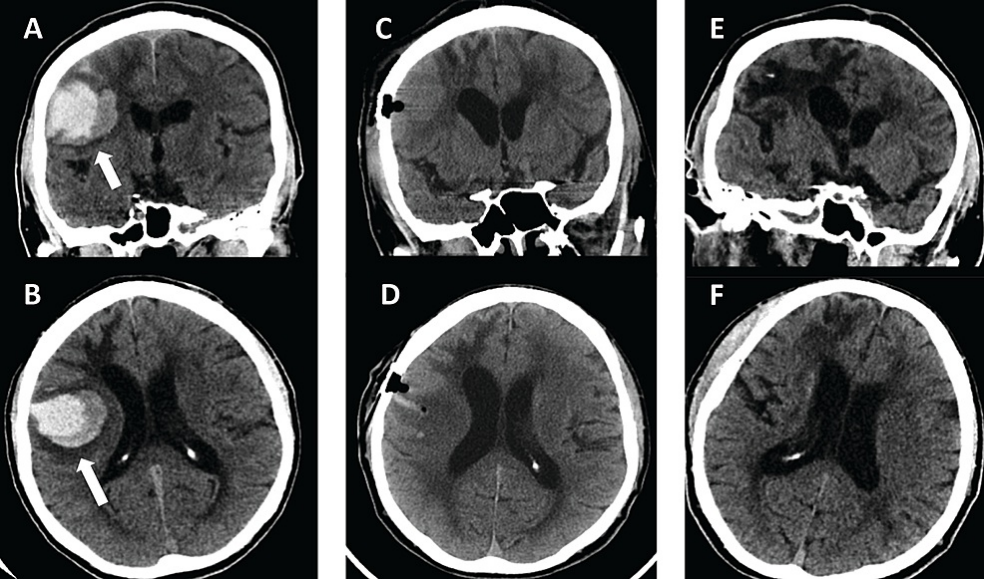
Case 2 - initial pre- and postprocedure and follow-up imaging. Preprocedure second admission non-contrast coronal (A) and axial (B) head computed tomography (CT) demonstrates a 42.5 mL right frontal hemorrhage (arrow). Postprocedure coronal (C) and axial (D) non-contrast CT demonstrated 86.8% evacuation with remaining hematoma measuring 5.6 mL. Coronal (E) and axial (F) follow-up imaging at one year showed the expected evolution of gliosis and encephalomalacia following hemorrhage.

A 69-year-old right-handed man with hypertension presented with left arm weakness and headache for one hour. On neurological exam, he was somnolent but arousable, oriented, and followed simple commands with left hemiparesis for an NIHSS of 6. CT revealed a 42.5 mL left frontal lobe and capsulostriatal ICH (Figures 3A, 3B). CTA showed no abnormalities. He underwent endoscopic evacuation on hospital day 3, reducing postoperative ICH volume by 86.8%, from 42.5 mL to 5.6 mL (Figures 3C, 3D). He was discharged to inpatient rehabilitation. At two- and six-month follow-up visits, his mRS was 2 (Figures 3E, 3F).

**Figure 4:**
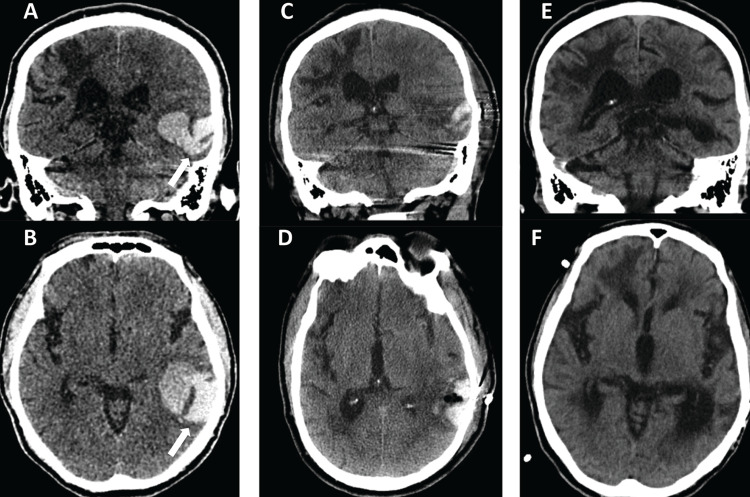
Case 2 - second admission pre- and postprocedure and follow-up imaging. Preprocedure second admission non-contrast coronal (A) and axial (B) head computed tomography (CT) demonstrated a 37.8 mL left posterior temporal hemorrhage (arrow). Postprocedure coronal (C) and axial (D) non-contrast CT demonstrated a 76.7% evacuation rate and the remaining hematoma measuring 8.8 mL. Coronal (E) and axial (F) follow-up imaging more than one year after the second hemorrhage showed the expected evolution of gliosis and encephalomalacia with hemorrhage resolution.

Approximately 20 months later, the patient presented with one day of word-finding difficulty. He did not follow commands, moved bilateral upper and lower extremities with mild residual left hemiparesis, and was NIHSS 15. CT revealed a 37.8 mL left posterior temporal ICH without IVH, causing 5.5 mm of midline shift (Figures 4A, 4B). CTA was negative for vascular abnormalities. Repeat head CT the following day showed increased hematoma volume while the NIHSS increased to 20, prompting an evacuation. Postoperative imaging showed 76.7% hematoma volume reduction, from 37.8mL to 8.8mL (Figure 4C, 4D). Postoperatively, the patient was eventually able to follow simple commands but remained aphasic, with baseline left motor weakness. At six-and twelve-month follow-up, his mRS was 4 (Figures 4E, 4F).

## Discussion

We describe two cases involving temporally distinct contralateral ICHs both treated with urgent endoscopic evacuation. Studies report an ICH recurrence rate of 1.8%-7.4% during the first year alone [17]. The ICH recurrences in our patients were unique in that they occurred in opposite hemispheres and were endoscopically evacuated on both occasions.

Previous studies have described long-term clinical outcomes of patients who received endoscopic evacuations, however, excluding those with recurrent or bilateral spontaneous ICH requiring subsequent intervention [7,12]. While our patients initially improved to near-functional independence after initial evacuations, they had significantly worse functional recoveries after the second presentation, despite small residual volumes (</=15 mL) after evacuation [6]. This could be related to the involvement of subcortical structures, secondary ICH locations in the left hemisphere, and/or diminished cognitive reserve after initial ICH.

Prior studies identify the deep cortical location as a poor indicator for long-term outcomes after ICH [13], showing thalamic and internal capsular involvement is associated with increased death or major disability [18]. While one patient in this report had ICH extending into deep cortical structures, the other developed radiographic evidence of gliosis and encephalomalacia in the deeper capsulostriatal region which possibly contributed to their outcomes.

Interestingly, no such association has been found with ICH laterality [19]. One potential reason could be that mRS does not explicitly consider lateralized cortical functions when determining functional scores. Instead, our patients’ poor recoveries could be due to the poorer baseline functional scores that both patients had before their second ICHs (mRS 2) compared to their initial ICH (mRS 0).

Lower preoperative functional scores have been shown to predict worse postoperative outcomes in ICH patients [12], possibly reflecting increased frailty and lower neurocognitive reserve to handle the stresses of additional ICH surgery. Previous studies have also demonstrated pre-ICH cognitive impairment to be an independent predictor of worse outcomes following ICH [20]. Indeed, evaluating cognitive decline is also important in assessing baseline functional status, as the cognitive decline is more prevalent in ICH patients compared to the general population, possibly due to the ICH cortical lesions or other comorbidities often present in these patients.

## Conclusions

Our case presentations demonstrate the functional decline experienced following bilateral ICH evacuation, after an initially reassuring outcome with the first surgery. This difference in outcome between the two surgeries is likely due to lower preoperative functional and cognitive baseline status, bilateral cortical pathology, and subcortical involvement.
